# Effect of organic mineral supplementation in reducing oxidative stress in Holstein calves during short-term heat stress and recovery conditions

**DOI:** 10.1186/s40104-023-00961-x

**Published:** 2023-12-22

**Authors:** A-Rang Son, Seon-Ho Kim, Mahfuzul Islam, Michelle Miguel, Ye Pyae Naing, Sang-Suk Lee

**Affiliations:** 1 Ruminant Nutrition and Anaerobe Laboratory, Department of Animal Science and Technology, Sunchon National University, Suncheon, 57922 Korea; 2 Department of Microbiology and Parasitology, Sher-e-Bangla Agricultural University, Dhaka, 1207 Bangladesh

**Keywords:** Antioxidant status, Heat stress, Holstein bull calves, Organic mineral supplementation, Oxidative stress

## Abstract

**Background:**

This study investigated the effects of inorganic and organic minerals on physiological responses, oxidative stress reduction, and rumen microbiota in Holstein bull calves (123.81 ± 9.76 kg; 5 months old) during short-term heat stress (HS) and recovery periods. Eight Holstein calves were randomly assigned to four treatment groups: no mineral supplementation (Con), inorganic minerals (IM), organic minerals (OM), and high-concentration organic minerals (HOM) and two thermal environments (HS and recovery) using 4 × 2 factorial arrangement in a crossover design of four periods of 35 d. Calves were maintained in a temperature-controlled barn. The experimental period consisted of 14 d of HS, 14 d of recovery condititon, and a 7-d washing period.

**Results:**

Body temperature and respiration rate were higher in HS than in the recovery conditions (*P* < 0.05). Selenium concentration in serum was high in the HOM-supplemented calves in both HS (90.38 μg/dL) and recovery periods (102.00 μg/dL) (*P* < 0.05). During the HS period, the serum cortisol was 20.26 ng/mL in the HOM group, which was 5.60 ng/mL lower than in the control group (*P* < 0.05). The total antioxidant status was the highest in the OM group (2.71 mmol Trolox equivalent/L), followed by the HOM group during HS, whereas it was highest in the HOM group (2.58 mmol Trolox equivalent/L) during the recovery period (*P* < 0.05). Plasma malondialdehyde and HSP70 levels were decreased by HOM supplementation during the HS and recovery periods, whereas SOD and GPX levels were not significantly affected (*P* > 0.05). The principal coordinate analysis represented that the overall rumen microbiota was not influenced by mineral supplementation; however, temperature-induced microbial structure shifts were indicated (PERMANOVA: *P* < 0.05). At the phylum level, Firmicutes and Actinobacteria decreased, whereas Fibrobacteres, Spirochaetes, and Tenericutes increased (*P* < 0.05), under HS conditions. The genus *Treponema* increased under HS conditions, while *Christensenella* was higher in recovery conditions (*P* < 0.05).

**Conclusion:**

HOM supplementation during HS reduced cortisol concentrations and increased total antioxidant status in Holstein bull calves, suggesting that high organic mineral supplementation may alleviate the adverse effects of HS.

## Background

The global temperature continues to rise; the hot and humid summer climate of Korea can cause heat stress (HS) in cattle. Beef cattle are particularly vulnerable to HS because they are unable to dissipate heat [1, 2]. Cattle exposed to HS consume less feed to prevent further increases in body temperature from metabolic heat generation [3, 4]. The loss of energy to maintain body temperature results in a lack of nutrients for growth and production [5], thereby maintaining cattle homeothermy at the expense of productivity and profitability [2]. Additionally, external indicators such as respiration rate and core body temperature are commonly used to determine HS damage [6, 7]. HS alters the gut microbiome, resulting in dysbiosis, impaired barrier and transport functions, and intestinal microstructures [8]. Moreover, at high temperatures, numerous reactive oxygen species (ROS), such as superoxide, hydrogen peroxide, and hydroxyl radicals, are generated in the body as by-products of the respiratory metabolic process [9]. ROS are capable to damage the antioxidant system in cattle and induce oxidative stress [10, 11]. At the cellular level, oxidative stress is an imbalance between oxidant species and antioxidants; cellular proteins, lipids, and DNA can be damaged by reactive oxygen, nitrogen, and other species formed from a compromised oxidative balance [12]. Various substances, such as minerals and vitamins, are known antioxidants that prevent oxidative cell damage; selenium, an antioxidant, affects the production of glutathione peroxidase, an antioxidant enzyme that prevents damage to cell membranes and peroxidation of fats and prevents cell function damage [13]. Zinc is also a component of superoxide dismutase, which scavenges superoxide, a component of ROS, in immune cells and affects protein and gene expression, animal reproduction, growth, and the immune system [14, 15]. Iron (Fe), a component of heme, is found primarily in hemoglobin and myoglobin; these proteins require iron as a cofactor for enzymes in the electron transport chain, specifically for myeloperoxidase, catalase, and cytochrome P-450. Iron deficiency results in hypochromic microcytic anemia due to failure in hemoglobin production, and the iron requirement is higher in young cattle than in mature cattle [16]. Iron also plays a beneficial role against oxidative stress in ruminants [17]. Traditionally, trace minerals have been supplied in the form of inorganic salts such as chlorides, sulphates, carbonates and oxides in ruminant diets, but their absorption rates are low and their excretion rates are high, which can cause environmental concerns [18, 19]. Supplying organic minerals can produce stable soluble molecules with high bioavailability and are better intestinal absorbed than inorganic minerals, due to the fact that organic form minerals are metal ions bound to organic substances such as peptides, amino acids, or polysaccharides and are then absorbed [20, 21]. Although the National Research Council (NRC) has advised the dose of the aforementioned minerals under normal circumstances [22], the ideal dose needed to counteract the negative effects of HS has not yet been established, which is a significant barrier for calf-raising. We hypothesized that high-concentration organic minerals (HOM) with antioxidant potential—as long as they don’t exceed the maximum permissible concentration advised by the NRC—can effectively and sustainably mitigate the negative effects of HS in calves during HS and the time afterwards. Our earlier study found that dairy steers with dietary higher mineral concentrations had lower levels of metabolic changes and oxidative damage related to HS [23]. Therefore, the objective of the present study was to evaluate the effect of inorganic and organic mineral supply on the rumen microbial community, blood oxidation state, and heat shock protein levels under stressful and non-stressful temperature conditions.

## Materials and methods

### Animal care

The study was conducted at the animal farm of the Sunchon National University (SCNU) and in the Ruminant Nutrition and Anaerobe Laboratory of the Department of Animal Science and Technology at SCNU, located in Jeonnam, South Korea. The handling of all experimental animals and relevant protocols were performed in accordance with the guidelines of the SCNU Institutional Animal Care and Use Committee (IACUC approval number: SCNU-IACUC-2020-06).

### Animals, experimental design, and diet

**Fig. 1:**
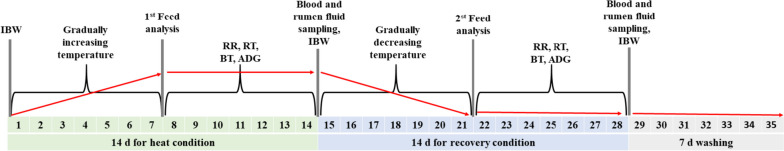
Timeline describing overall study design, data, and sample collection for each period. IBW, Initial body weight; RR, Respiration rate; RT, Rectal temperature; BT, Body temperature; ADG, Average daily gain

The experimental design was a 4-period crossover with a 4 × 2 factorial arrangement involving 4 treatment groups (Con, IM, OM, and HOM) and two thermal environments (HS and recovery). The experiment trial was conducted for 140 d (July 2021 to November 2021) with each period lasting 35 d, including 14 d of HS, 14 d of recovery (thermal neutral) condition and a 7-d washing period (Fig. 1). Eight Holstein bull calves (123.81 ± 9.76 kg; 5 months old) were used in the study. The calves were randomly distributed into four groups of 2 calves each to evaluate the four treatments. Calves were kept in individual pens in a temperature-controlled barn. Calves were fed once daily (0900 h) with Timothy hay and concentrate feed (CJ Feed & Care, Korea) separately at a ratio of 6:4 and had free access to water. The treatments included a control (without mineral supplementation), inorganic minerals (IM), organic minerals (OM), and high-concentration organic minerals (HOM), and the top dressing was performed at 0.4% of the basal diet (Table 1). Inorganic minerals used were ZnSO_4_ and MnSO_4_ (Seoan Chemtec Co., Ltd., Korea), FeSO_4_, CuSO_4_ and Na_2_SeO_3_ (TMC Co., Ltd., Korea), Ca(IO_3_)_2_ and CoSO_4_ (Innobatech Co., Ltd., Korea), while the organic minerals used were amino acid complex Zn, Mn, Fe, Cu, I, Co, and Se (Prochem Co., Ltd., Korea). The ingredients and chemical composition of the basal diet are listed in Table 2 which were formulate according to NRC [22]. Feed intake was determined daily by weekly collection of feed residues and ingredients. The chemical composition of the basal diet was analyzed using standard methods [24]. Neutral detergent fiber (NDF) and acid detergent fiber (ADF) contents were determined according to the protocols described by Van Soest et al. [25] and Van Soest [26], respectively.

**Table 1:** Composition of mineral supplementation

Ingredient composition	IM	OM	HOM
Zinc, mg/kg	50	50	100
Manganese, mg/kg	24	24	24
Iron, mg/kg	50	50	100
Copper, mg/kg	15	15	15
Iodine, mg/kg	0.8	0.8	0.8
Cobalt, mg/kg	0.4	0.4	0.4
Selenium, mg/kg	0.3	0.3	2

*IM* Inorganic minerals supplementation, *OM* Organic minerals supplementation, *HOM* Higher organic minerals supplementation

**Table 2:** Chemical composition of the Timothy hay and concentrate percentage

Item, % (as DM basis)	Timothy hay	Concentrate
DM (fresh basis)	91.11	89.62
Crude protein	8.96	17.92
Crude fiber	36.4	14.62
Crude fat	3.41	3.24
Crude ash	7.10	7.69
Calcium	0.22	1.29
Phosphorus	0.22	0.58
NDF	65.26	36.19
ADF	38.23	18.87

*DM* Dry matter, *NDF* Neutral detergent fiber, *ADF* Acid detergent fiber

**Fig. 2:**
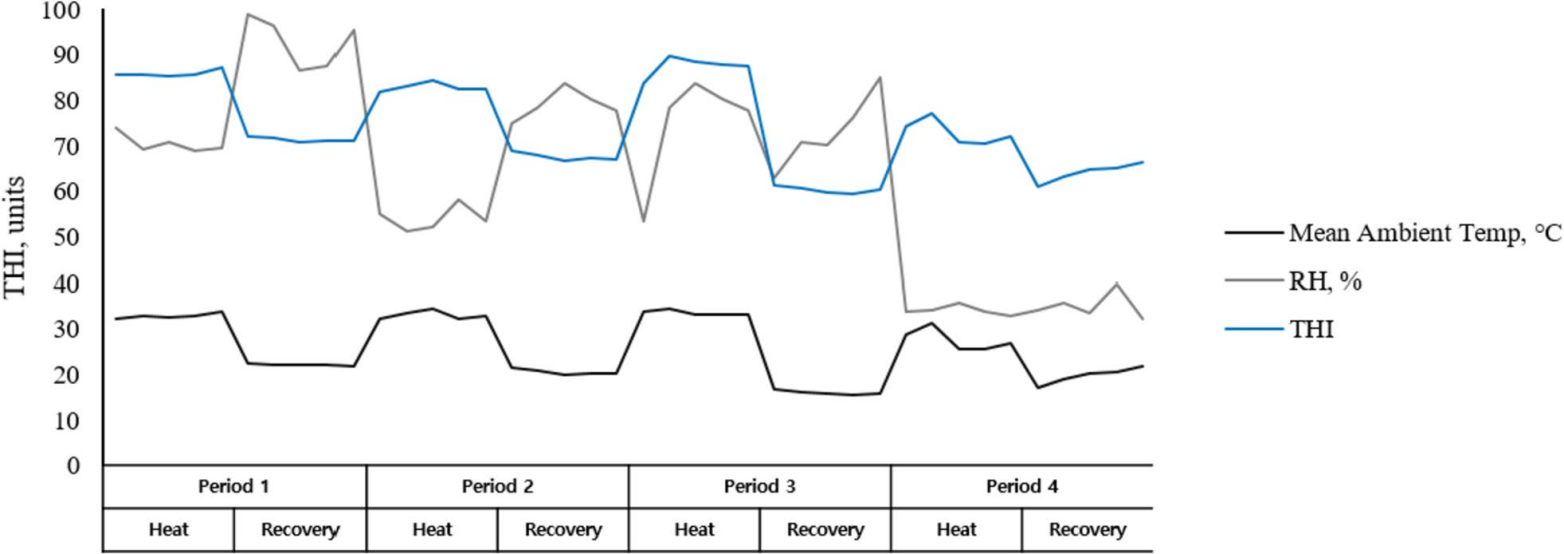
Recorded ambient temperature, relative humidity, and temperature humidity index during study periods. RH, relative humidity; THI, temperature humidity index

The ambient temperature (°C) and relative humidity (%) of the experimental shed were recorded throughout the experimental period using a Testo 174H Mini data logger (West Chester, PA, USA) and are shown in Fig. 2. The temperature humidity index (THI) was calculated as THI = (0.8 × ambient temperature) + [(% relative humidity/100) × (ambient temperature − 14.4)] + 46.4 [27].

### Sample collection and measurements

Body weight before feeding was measured on d 14 and 28 of each period, and 15 mL of blood was collected from the jugular vein of each calf 2 h after feeding. The collected blood was immediately transferred to 3 vacutainers (SSTTM II Advance, Trace Element serum, K2 EDTA; BD vacutainer®) of 5 mL each and centrifuged at 3,000 r/min for 15 min, and the serum and plasma samples were transferred into 2-mL microtubes and stored at −80 °C until analysis. After blood collection, rumen fluid samples were collected using a stomach tube, and pH was immediately measured using a pH meter (Seven CompactTM pH/Ion meter S220, Mettler Toledo, Switzerland). Simultaneously, the rumen fluid was transported to the laboratory using dry ice and stored at −80 °C until ammonia nitrogen (NH_3_-N), volatile fatty acids (VFAs), and rumen microbiota could be analyzed. In addition, the respiration rate, rectal temperature, and body temperature were measured twice a day (09:00 and 15:00) for three consecutive days starting from d 11 and 25 in the HS and recovery periods, respectively. The respiration rate was converted to the respiration rate per minute by multiplying by 2 after observing the movement of the flank for 30 s. The rectal temperature was recorded 30 s after the digital thermometer (WPT-1; CAS, Korea; accuracy ± 1 ºC) was inserted into the rectum, and the body temperature was measured using an infrared thermometer (SO-20-01; Apollo, Korea; accuracy ± 0.2 ºC).

### Sample analysis

#### Analysis of serum biochemistry, mineral concentrations, cortisol, total oxidant status, total antioxidant status, and oxidative stress index

Serum calcium (Ca), magnesium (Mg), phosphorus (P), blood urea nitrogen (BUN), total protein (TP), aspartate aminotransferase (AST), total bilirubin (TBIL), and cholesterol (CHOL) levels were analyzed using a biochemical analyzer (IDEXX Catalyst One, USA). Serum glucose levels were analyzed using a portable glucose test meter (FreeStyle Optium Neo H Blood Glucose and Ketone System; Abbott Diabetes Care Ltd., UK). Serum concentrations of Zn and Se were analyzed using an inductively coupled plasma mass spectrometry (ICP-MS) system (ELAN DRCe, PerkinElmer, Germany), and Fe was analyzed using a Cobas 8000 (Roche, Germany). Serum cortisol levels were measured using a bovine cortisol enzyme-linked immunosorbent assay (ELISA) kit (cat. MBS2608983, Mybiosource), and total oxidant status (TOS) and total antioxidant status (TAS) were measured using the Rel Assay kit (Rel Assay Diagnostics, Turkey) following the manufacturer’s instructions. The oxidative stress index (OSI) was calculated according to the formula OSI = [(TOS, mol/L)/(TAS, mmol Trolox equivalent/L) × 100] [28].

#### Analysis of plasma superoxide dismutase, glutathione peroxidase, malondialdehyde, and heat shock proteins

Plasma superoxide dismutase (SOD, Item # 706002) and glutathione peroxidase (GPX, Item # 703102) levels were measured using an ELISA kit (Cayman Chemical Company, Ann Arbor, USA) following the manufacturer’s instructions, and malondialdehyde (MDA) was measured using an MDA Assay kit (ab238537, Abcam) according to the manufacturer’s instructions. HSP levels (HSP27, HSP70, and HSP90) were measured using MyBioSource (San Diego, CA, USA) bovine ELISA kits (MBS011935, MBS2882245, and MBS094979) following the manufacturer’s instructions.

#### Analysis of NH_3_-N and VFA concentrations

The NH_3_-N concentration was measured colorimetrically using a Libra S22 spectrophotometer (CB40FJ; Biochrom Ltd., Cambourne, UK), following the protocol described by Chaney and Marbach [29]. VFA concentration was measured according to the method described by Han et al. [30]. and Tabaru et al. [31] using high-performance liquid chromatography (HPLC; Agilent Technologies 1200 series, Waldbronn, Germany). A UV detector (set at 210 nm and 220 nm), METACARB87H column (Varian, Palo Alto, CA, USA), and buffered solvent (0.00425 mol/L H_2_SO_4_) at a flow rate of 0.6 mL/min were used to perform HPLC.

### DNA extraction and PCR amplification and 16S rRNA amplicon sequencing

All rumen fluid samples were sent to Macrogen, Inc. (Seoul, Korea) for DNA extraction and metataxonomic analysis of the rumen microbiota. Briefly, DNA was extracted using a DNeasy PowerSoil kit (Qiagen, Hilden, Germany), following the manufacturer’s protocol [32]. The quality and quantity of the DNA were assessed using PicoGreen and NanoDrop, respectively. Sequencing libraries were prepared according to the Illumina 16S Metagenomic Sequencing Library protocols to amplify the V3 and V4 regions. The PCR assay was performed with 2 ng of gDNA, 1× reaction buffer, 1 nmol/L of dNTP mix, 500 nmol/L of each of the universal F/R PCR primers, and 2.5 U of Herculase II fusion DNA polymerase (Agilent Technologies, Santa Clara, CA, USA). The cycle condition for 1^st^ PCR was 3 min at 95 °C for heat activation, and 25 cycles of 30 s at 95 °C, 30 s at 55 °C, and 30 s at 72 °C, followed by a 5 min final extension at 72 °C. The universal primer pair with Illumina adapter overhang sequences used for the first amplification were as follows: V3-F: 5′-TCG TCG GCA GCG TCA GAT GTG TAT AAG AGA CAG CCT ACG GGN GGC WGC AG-3′, V4-R: 5′-GTC TCG TGG GCT CGG AGA TGT GTA TAA GAG ACA GGA CTA CHV GGG TAT CTA ATC C-3′. AMPure beads (Agencourt Bioscience, Beverly, MA, USA) were used to purify the products of the first and second PCR. Individual amplicon libraries were normalized after quantification using PicoGreen. They were then size-verified using a TapeStation DNA ScreenTape D1000 (Agilent Technologies), pooled at an equimolar ratio, and sequenced on a MiSeq system (Illumina, San Diego, CA, USA) using a 2 × 300 bp kit.

### Sequence data processing and metataxonomic analysis

After sequencing, the raw Illumina MiSeq data were classified by sample using an index sequence, and a paired-end FASTQ file was generated for each sample. Sequencing adapter and F/R primer sequences of the target gene region were removed using Cutadapt (v3.2) [33].

The DADA2 (v1.18.0) [34] package of R (v4.0.3) was used for error correction in the amplicon sequencing process. For paired-end reads, the forward sequence (Read1) and reverse sequence (Read2) were cut to 250 bp and 200 bp, respectively, and sequences with expected errors of two or more were excluded. An error model was established for each batch to remove noise from each sample. After assembling the paired-end sequence corrected for sequencing errors into one sequence, the chimeric sequence was removed using the DADA2 Consensus method to form amplicon sequence variants (ASVs). In addition, for a comparative analysis of the microbial community, the QIIME (v1.9) [35] program was used for normalization by applying subsampling based on the number of reads of the sample with the minimum number of reads among all samples.

For each ASV sequence, BLAST + (v2.9.0) [36] was performed using the reference database (DB; NCBI 16S Microbial DB), and the taxonomic information for the organism of the subject with the highest similarity was assigned. At this time, if the query coverage of the best-hit matching in the DB was less than 85% or the identity of the matched area was less than 85%, taxonomy information was not allocated.

Various microbial communities were comparatively analyzed using QIIME with the aforementioned ASVs abundances and taxonomic information. To analyze beta diversity indices, we used principal coordinate analysis (PCoA) based on both unweighted and weighted UniFrac distances for visualization [37]. Before analysis, we assessed the homogeneity of multivariate dispersion among treatments and groups using permutational multivariate analysis of dispersion (PERMDISP) [38]. Subsequently, a permutational analysis of variance (PERMANOVA) was performed to detect any potential differences in the microbial community structures between the minerals and temperatures. All procedures, including PCoA, PERMDISP, and PERMANOVA, were conducted using the vegan package in R software (v4.0.3).

### Statistical analysis

Before analysis, the homoscedasticity and normality of the distribution assumptions were tested. Growth performance, respiration rate, body temperature, blood parameters, and rumen fermentation parameters were analyzed by repeated measures using MIXED procedure of the SAS statistical package (version 9.4; SAS Institute Inc., Cary, NC, USA) with an autoregressive covariance structure. The model included the main fixed effects of temperature, minerals, and interactions between the two variables, whereas the effect of each calf was considered random [39]. Statistical significance was set at *P* < 0.05 unless otherwise stated. Following a significant effect, Duncan’s post-hoc test was performed to assess the differences between the means.

## Results

### Growth performance, body temperature, and respiration rate

**Fig. 3:**
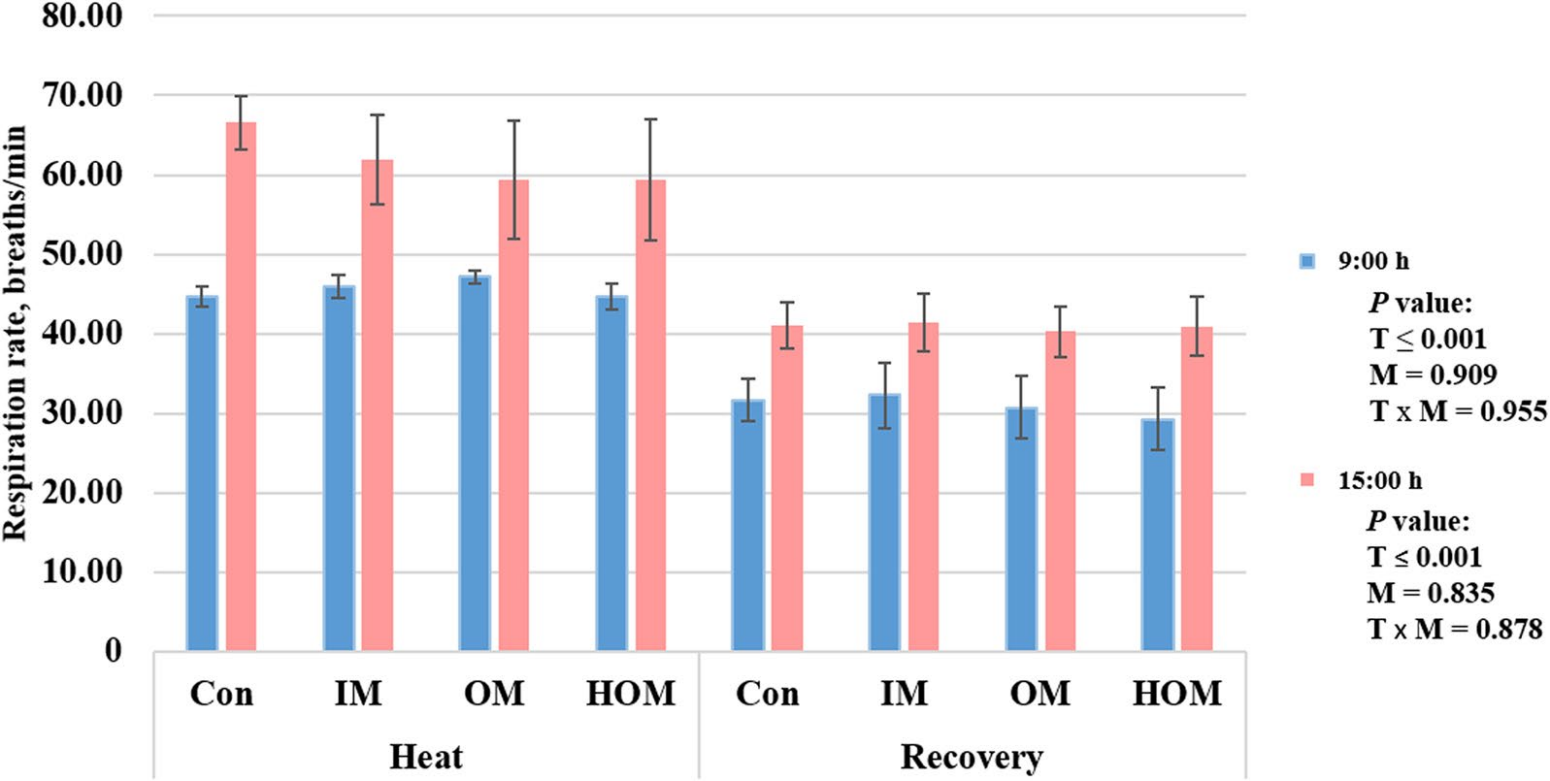
Respiration rate of Holstein calves supplemented with inorganic and organic minerals at different temperatures. Con: Basal diet (without mineral supplementation), IM: Basal diet + inorganic minerals supplementation, OM: Basal diet + organic minerals supplementation, HOM: Basal diet + higher organic minerals supplementation

The growth performance of Holstein calves supplemented with inorganic and organic minerals at different temperatures is shown in Table 3. The average daily gain (ADG; kg), dry matter intake (DMI; kg/d), and FE were not significantly different between the treatment groups. The body temperatures and respiration rates of Holstein calves supplemented with inorganic and organic minerals at different temperatures are presented in Table 4 and Fig. 3. Body temperature was higher in the HS condition than in the recovery condition for all parameters (rectal, flank, rump, perineum, belly, back, and thigh) at 15:00 h (*P* < 0.05). The respiration rate was also higher in the HS condition than during the recovery period at both time points (*P* < 0.05).

**Table 3:** Growth performance of Holstein calves supplemented with inorganic and organic minerals at different temperatures

Parameters	Heat				Recovery				SEM^5^	Mixed *P* value^6^		
	Con^1^	IM^2^	OM^3^	HOM^4^	Con^1^	IM^2^	OM^3^	HOM^4^		Temperature	Minerals	T × M
IBW, kg	147.20	147.61	146.35	148.14	153.47	154.59	153.81	155.81	11.576	0.395	0.998	0.999
FBW, kg	153.47	154.59	153.81	155.81	161.88	161.69	160.81	162.06	12.037	0.408	0.999	0.999
ADG, kg	0.63	0.70	0.75	0.77	0.84	0.71	0.70	0.63	0.118	0.921	0.991	0.538
DMI, kg/d	4.83	4.74	4.87	4.86	5.08	4.95	5.11	5.10	0.279	0.247	0.950	0.999
FE	0.13	0.15	0.15	0.16	0.16	0.14	0.14	0.12	0.023	0.611	0.984	0.555

*IBW* Initial body weight, *FBW* Final body weight, *ADG* Average daily gain, *DMI* Dry matter intake, *FE* Feed efficiency

^1^Con: Basal diet (without mineral supplementation)

^2^IM: Basal diet + inorganic minerals supplementation

^3^OM: Basal diet + organic minerals supplementation

^4^HOM: Basal diet + higher organic minerals supplementation

^5^Standard error of means

^6^*P* value obtained from the mixed procedure of SAS with temperature effect (B), mineral effect (M), and interaction effect between temperature and minerals (T × M). A *P* value less than 0.05 indicates that the results are statistically significant

**Table 4:** Body temperature of Holstein calves supplemented with inorganic and organic minerals at different temperatures

Time	Parameters	Heat				Recovery				SEM^5^	Mixed *P* value^6^		
		Con^1^	IM^2^	OM^3^	HOM^4^	Con^1^	IM^2^	OM^3^	HOM^4^		Temperature	Minerals	T × M
9:00 h	Rectal	39.82	39.83	39.88	39.71	39.61	39.65	39.73	39.65	0.114	0.097	0.787	0.934
	Flank	36.23	36.15	36.37	36.09	35.10	35.30	35.04	35.18	0.614	0.024	0.999	0.980
	Rump	36.03	36.01	36.00	35.89	34.70	34.71	35.13	34.82	0.742	0.042	0.991	0.989
	Perineum	36.83	36.85	36.81	36.75	36.32	36.30	36.31	36.36	0.277	0.049	0.999	0.996
	Belly	35.94	35.85	36.01	36.02	35.09	35.29	35.13	35.25	0.549	0.062	0.997	0.992
	Back	36.23	35.72	35.98	36.10	35.28	34.75	34.95	34.69	0.666	0.033	0.892	0.985
	Thigh	36.01	36.10	36.05	35.98	35.08	35.21	35.38	35.61	0.412	0.025	0.945	0.904
15:00 h	Rectal	40.33	40.29	40.41	40.33	40.04	40.05	40.07	39.85	0.173	0.029	0.905	0.943
	Flank	36.55	36.85	36.79	36.87	35.67	35.53	35.26	35.57	0.544	0.004	0.985	0.945
	Rump	36.60	36.61	36.70	36.55	35.25	35.46	35.19	35.26	0.590	0.005	0.996	0.992
	Perineum	37.91	37.67	37.80	37.77	36.59	36.77	36.55	36.73	0.452	0.004	0.998	0.973
	Belly	36.58	36.55	36.90	36.42	35.63	35.54	35.40	35.36	0.430	0.001	0.938	0.916
	Back	36.75	36.75	36.71	36.76	35.56	35.47	35.30	35.46	0.522	0.002	0.993	0.997
	Thigh	36.69	36.74	36.88	36.88	36.09	36.01	35.97	35.98	0.273	0.001	0.996	0.934

^1^Con: Basal diet (without mineral supplementation)

^2^IM: Basal diet + inorganic minerals supplementation

^3^OM: Basal diet + organic minerals supplementation

^4^HOM: Basal diet + higher organic minerals supplementation

^5^Standard error of the mean

^6^*P* value received from the mixed procedure of SAS with temperature effect (B), mineral effect (M), and interaction effect between temperature and minerals (T × M). A *P* value less than 0.05 indicates that the results are statistically significant

### Serum biochemistry, mineral concentrations, cortisol, TOS, TAS, and OSI

**Fig. 4:**
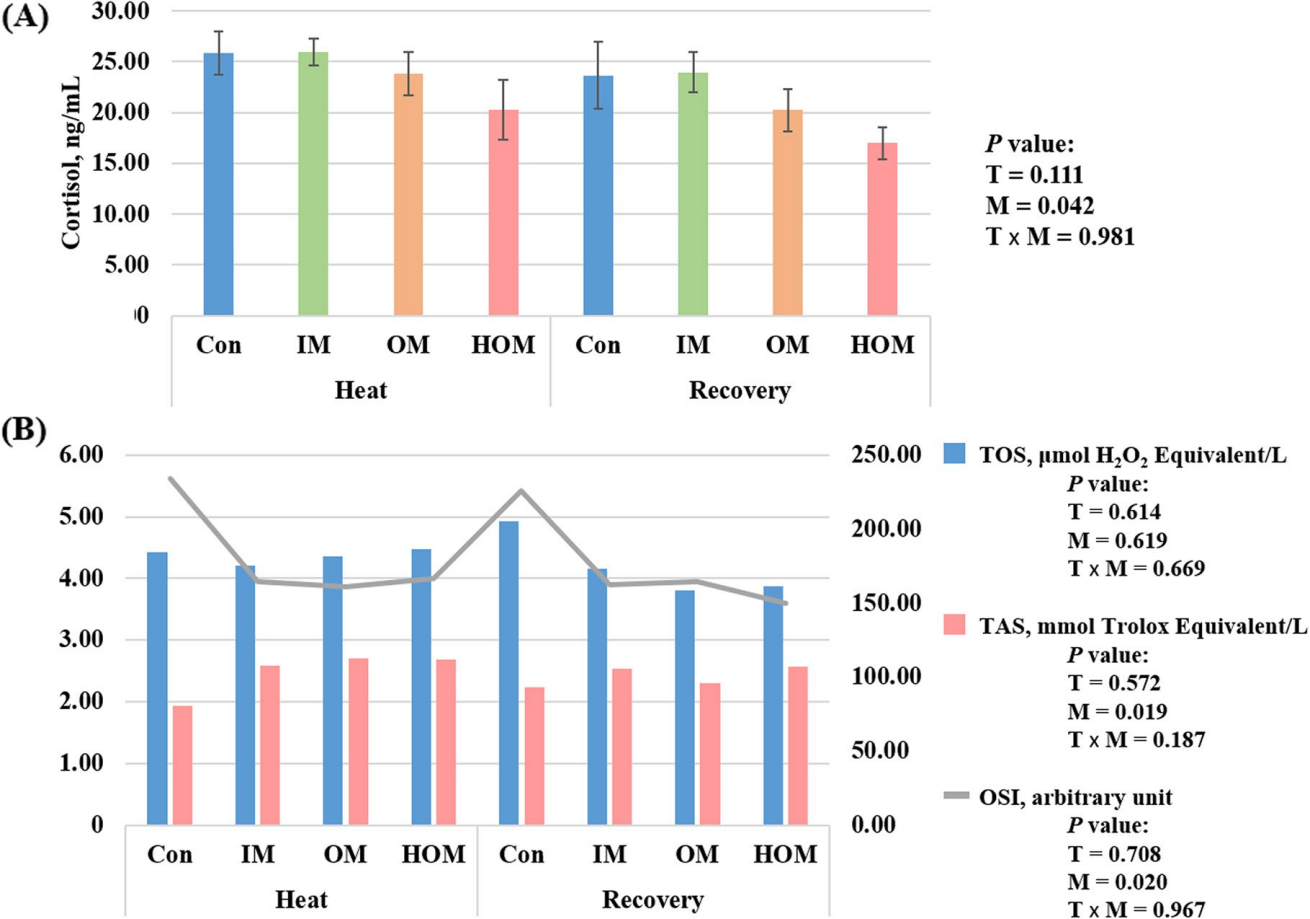
Serum cortisol (**A**) and total oxidant status (TOS), total antioxidant status (TAS), and oxidative stress index (OSI) (**B**) of Holstein calves supplemented with inorganic and organic minerals at different temperatures. Con: Basal diet (without mineral supplementation), IM: Basal diet + inorganic minerals supplementation, OM: Basal diet + organic minerals supplementation, HOM: Basal diet + higher organic minerals supplementation

The serum biochemistry and mineral concentrations of Holstein calves supplemented with inorganic and organic minerals at different temperatures are presented in Table 5. None of the tested serum biochemical parameters were significantly different between the different temperature and treatment groups. Serum Se concentration was higher in the recovery condition than in the HS condition (*P* < 0.05), and in the HOM group, it was the highest at 90.38 μg/dL and 102.00 μg/dL in the heat and recovery conditions, respectively (*P* < 0.05). The results for serum cortisol, TOS, TAS, and OSI in Holstein calves supplemented with inorganic and organic minerals at different temperatures are presented in Fig. 4. The serum cortisol concentration was 20.26 ng/mL in the HOM group in the HS condition, 5.60 ng/mL lower than that in the control group (*P* < 0.05). The TAS was the highest with 2.71 mmol Trolox equivalent/L in the OM group, followed by the HOM group under HS conditions and was the highest at 2.58 mmol Trolox equivalent/L in the HOM group under the recovery conditions (*P* < 0.05). The OSI was lower in the mineral-supplemented groups than in the Con in both periods, and the lowest OSI was observed in the HOM group (149.61) under recovery conditions (*P* < 0.05).

**Table 5:** Serum biochemistry and mineral concentrations of Holstein calves supplemented with inorganic and organic minerals at different temperatures

Parameters	Heat				Recovery				SEM^5^	Mixed *P* value^6^		
	Con^1^	IM^2^	OM^3^	HOM^4^	Con^1^	IM^2^	OM^3^	HOM^4^		Temperature	Minerals	T × M
Glucose, mg/dL	69.25	68.75	70.88	68.50	65.75	67.00	67.13	68.75	4.661	0.532	0.989	0.974
BUN, mg/dL	6.38	6.88	7.13	6.25	6.13	7.25	6.25	6.38	1.127	0.847	0.882	0.951
PHOS, mg/dL	7.55	7.55	7.40	7.41	7.75	7.85	7.35	7.61	0.273	0.438	0.683	0.941
Ca, mg/dL	9.24	9.18	9.26	9.08	9.35	9.21	9.43	9.24	0.159	0.312	0.640	0.977
Mg, mg/dL	2.00	2.01	1.95	2.03	2.01	2.01	2.06	2.01	0.033	0.277	0.989	0.270
Total P, g/dL	6.81	6.76	6.65	6.69	6.84	6.76	6.90	6.79	0.126	0.308	0.918	0.761
AST, U/L	81.00	80.50	81.50	82.50	83.63	87.75	88.00	87.75	3.320	0.046	0.871	0.925
TBIL, mg/dL	0.25	0.30	0.31	0.24	0.21	0.25	0.30	0.21	0.022	0.071	0.006	0.869
CHOL, mg/dL	78.25	72.63	73.13	75.00	88.13	75.88	84.88	81.75	7.930	0.198	0.771	0.961
Iron, μg/dL	137.50	136.75	140.00	146.75	144.00	145.75	141.88	142.75	7.728	0.573	0.956	0.869
Zn, μg/dL	97.00	100.00	95.63	102.38	100.63	103.38	106.00	106.13	4.764	0.186	0.798	0.901
Se, μg/dL	67.00	74.38	73.38	90.38	70.75	74.75	77.25	102.00	3.198	0.047	< 0.001	0.395

^1^Con: Basal diet (without mineral supplementation)

^2^IM: Basal diet + inorganic minerals supplementation

^3^OM: Basal diet + organic minerals supplementation

^4^HOM: Basal diet + higher organic minerals supplementation

^5^Standard error of means

^6^*P* value obtained from the mixed procedure of SAS with temperature effect (B), mineral effect (M), and interaction effect between temperature and minerals (T × M). A *P* value less than 0.05 indicates that the results are statistically significant

### Plasma SOD, GPX, MDA, and HSPs

The results of plasma SOD, GPX, MDA, and HSPs in Holstein calves supplemented with inorganic and organic minerals at different temperatures are presented in Table 6. The plasma SOD and GPX concentrations were numerically lower, whereas HSPs were numerically higher in the mineral-supplemented groups than in the Con group under both heat and recovery conditions (*P* > 0.05). The HOM group under heat and recovery conditions showed lower plasma MDA and HSP70 levels and higher SOD and GPX activities (*P* > 0.05).

**Table 6:** Plasma SOD, GPX, MDA, and HSPs of Holstein calves supplemented with inorganic and organic minerals at different temperatures

Parameters	Heat				Recovery				SEM^5^	Mixed *P* value^6^		
	Con^1^	IM^2^	OM^3^	HOM^4^	Con^1^	IM^2^	OM^3^	HOM^4^		Temperature	Minerals	T × M
SOD, U/mL	33.38	35.72	33.78	35.69	32.03	33.05	33.17	34.08	0.990	0.115	0.368	0.892
GPX activity, nmol/min/mL	34.70	35.98	36.72	36.21	34.14	36.05	36.21	37.41	3.469	0.984	0.920	0.994
MDA, μmol/L	1.53	1.50	1.37	1.24	1.60	1.59	1.44	1.29	0.144	0.549	0.263	0.999
HSP27kDa, pg/mL	742.57	691.42	663.04	667.63	833.49	806.98	715.89	753.28	60.783	0.077	0.451	0.972
HSP70kDa, ng/mL	0.24	0.22	0.22	0.21	0.25	0.22	0.20	0.20	0.017	0.701	0.169	0.796
HSP90kDa, ng/mL	6.41	5.90	5.98	5.58	6.14	5.28	5.89	5.38	0.678	0.542	0.620	0.983

*SOD* Superoxide dismutase, *GPX* Glutathione peroxidase, *MDA* Malondialdehyde, *HSP* Heat shock protein

^1^Con: Basal diet (without mineral supplementation)

^2^IM: Basal diet + inorganic minerals supplementation

^3^OM: Basal diet + organic minerals supplementation

^4^HOM: Basal diet + higher organic minerals supplementation

^5^Standard error of means

^6^*P* value obtained from the mixed procedure of SAS with temperature effect (B), mineral effect (M), and interaction effect between temperature and minerals (T × M). A *P* value less than 0.05 indicates that the results are statistically significant

### Rumen fermentation characteristics and composition of rumen microbiota

**Fig. 5:**
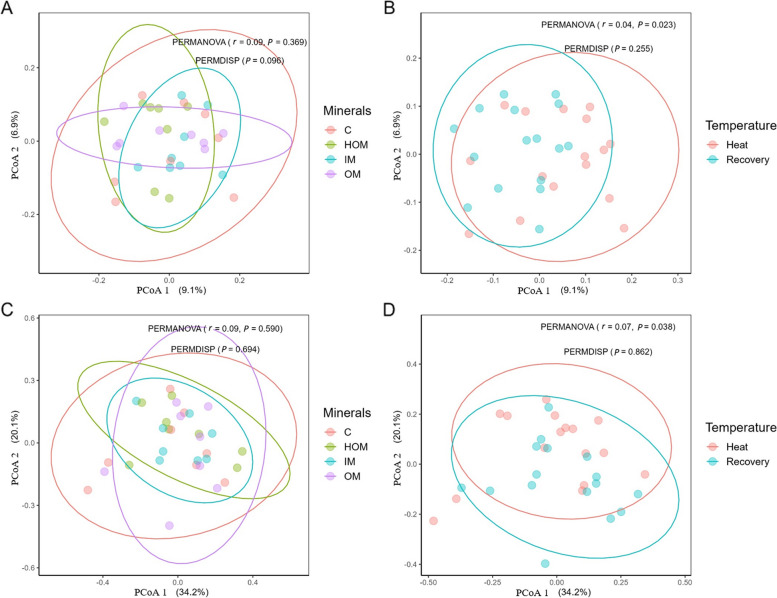
PCoA of rumen microbiota of Holstein calves supplemented with inorganic and organic minerals at different temperatures. **A** Unweighted UniFrac distances (minerals). **B** Unweighted UniFrac distances (temperature). **C** Weighted UniFrac distances (minerals). **D** Weighted UniFrac distances (temperature). Con (C): Basal diet (without mineral supplementation), IM: Basal diet + inorganic minerals supplementation, OM: Basal diet + organic minerals supplementation, HOM: Basal diet + higher organic minerals supplementation

**Fig. 6:**
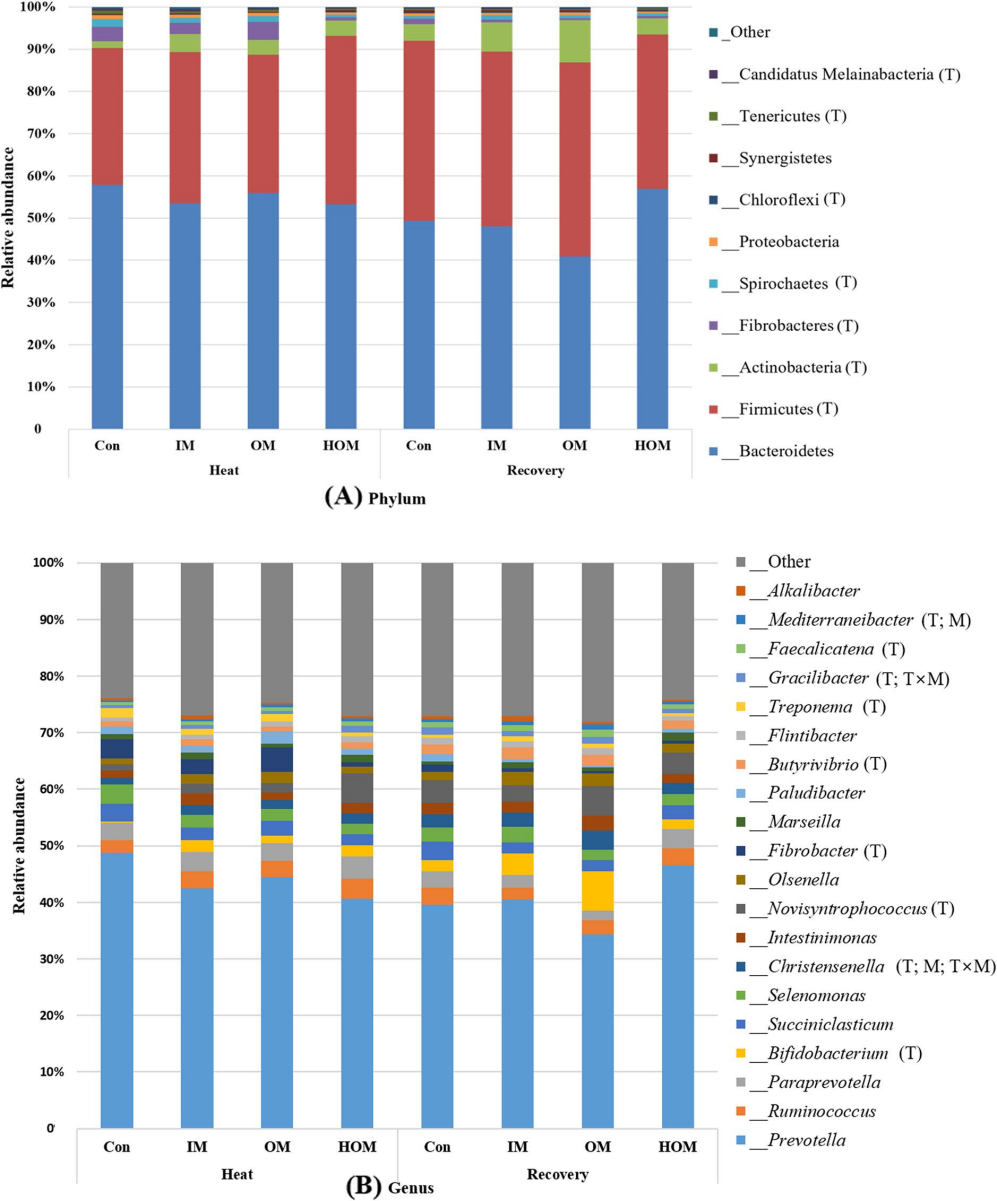
Rumen bacterial abundance of Holstein calves supplemented with inorganic and organic minerals at different temperatures. **A** Phylum level. **B** Genus level. Con: Basal diet (without mineral supplementation), IM: Basal diet + inorganic minerals supplementation, OM: Basal diet + organic minerals supplementation, HOM: Basal diet + higher organic minerals supplementation

The rumen fermentation characteristics of Holstein calves supplemented with inorganic and organic minerals at different temperatures are presented in Table 7. No significant differences were observed in pH, NH_3_-N, or total VFA among the different temperatures and treatment groups. Figure 5A and B show the beta diversity results. Individuals were projected onto the first two dimensions of PCoA, which accounted for 9.1% and 6.9% of the variability in unweighted UniFrac distances, respectively. Similarly, the first two dimensions of the PCoA accounted for 34.1% and 20.1% of the variability in the weighted UniFrac distances observed between the samples (Fig. 5C and D). Regardless of the factors investigated (temperature and minerals) or the distance matrix utilized (unweighted or weighted UniFrac), no significant differences in the individual dispersions were observed (PERMDISP: *P* > 0.05). Moreover, the PERMANOVA procedure failed to detect substantial differences (*P* > 0.05) between the minerals using either unweighted or weighted UniFrac distances. Conversely, PERMANOVA revealed a significant influence of temperature on the overall microbial structure, based on both unweighted (*P* = 0.023) and weighted (*P* = 0.038) UniFrac distances. The ruminal bacterial abundances in Holstein calves supplemented with inorganic and organic minerals at different temperatures are presented in Fig. 6. Upon evaluating the composition of the rumen microbiota, at the phylum level, Bacteroidetes (accounting for 40.86% to 57.87%) and Firmicutes (32.40% to 45.96%) were the two major bacterial taxa among all treatment groups. Firmicutes and Actinobacteria decreased, whereas Fibrobacteres, Spirochaetes, and Tenericutes increased under HS conditions (Fig. 6A). At the genus level, *Treponema* increased during HS, whereas *Christensenella* increased during the recovery period (Fig. 6B).

**Table 7:** Rumen fermentation characteristics of Holstein calves supplemented with inorganic and organic minerals at different temperatures

Parameters	Heat				Recovery				SEM^5^	Mixed *P* value^6^		
	Con^1^	IM^2^	OM^3^	HOM^4^	Con^1^	IM^2^	OM^3^	HOM^4^		Temperature	Minerals	T × M
pH	6.23	6.26	6.21	6.40	6.46	6.32	6.29	6.37	0.069	0.109	0.334	0.438
NH_3_-N, mg/dL	4.96	4.34	4.11	3.51	4.95	4.65	3.97	3.48	0.752	0.956	0.295	0.993
Total VFA, mmol/L	113.02	109.28	111.48	106.05	112.28	105.02	111.06	106.75	6.936	0.820	0.788	0.988
Acetate, mmol/L	59.89	60.17	62.85	60.48	60.81	58.46	60.26	60.96	3.077	0.748	0.909	0.931
Propionate, mmol/L	33.76	30.69	32.20	28.55	33.44	29.76	32.99	29.81	3.081	0.933	0.528	0.987
Butyrate, mmol/L	19.37	18.42	16.43	17.02	18.04	16.80	17.80	15.97	1.786	0.664	0.767	0.882
A:P	1.81	1.99	2.06	2.17	1.83	1.97	1.93	2.05	0.152	0.606	0.439	0.961
Acetate, %	53.40	55.39	56.68	57.15	54.28	55.60	54.57	57.08	1.450	0.806	0.257	0.783
Propionate, %	29.64	27.98	28.66	26.67	29.72	28.38	29.31	27.91	1.453	0.617	0.531	0.988
Butyrate, %	16.96	16.63	14.66	16.18	16.00	16.02	16.12	15.01	1.237	0.753	0.829	0.775

*A:P* Acetate:propionate, *NH*_*3*_*-N* Ammonia–nitrogen, *VFA* Volatile fatty acids

^1^Con: Basal diet (without mineral supplementation)

^2^IM: Basal diet + inorganic minerals supplementation

^3^OM: Basal diet + organic minerals supplementation

^4^HOM: Basal diet + higher organic minerals supplementation

^5^Standard error of means

^6^*P* value obtained from the mixed procedure of SAS with temperature effect (B), mineral effect (M), and interaction effect between temperature and minerals (T × M). A *P* value less than 0.05 indicates that the results are statistically significant

## Discussion

Cattle exposed to HS show reduced feed intake, increased water intake, and changes in endocrine status, resulting in decreased body weight and average daily gain, and worsening body condition due to increased maintenance requirements [40]. However, non-significant variations in ADG (kg) and FE were observed among the treatment groups, which may be due to the short-term HS conditions used in this study. Body temperature and respiration rate (RR) were unaffected by mineral supply but increased under HS conditions according to the change in THI. This is similar to the study in which rectal temperature and RR were considered visible physiological indicators for determining the thermal regulation response of dairy cows to heat stress and that they are altered due to high environmental temperatures [41, 42].

Among these minerals, selenium, iron, and zinc play important roles in animal growth and health by being involved in the antioxidant defense system [17, 43, 44]. Increased lipid peroxidation under stress conditions stimulates the stress axis to increase cortisol concentrations [45, 46]. In this study, the supply of organic minerals (OM and HOM groups) was beneficial in reducing cortisol levels and oxidative stress index during short-term HS.

The MDA concentration is an indicator of lipid peroxidation [47], and antioxidant enzymes, such as SOD and GPX, can be synthesized in the body to remove ROS generated during HS [48, 49]. HSP70 expression has been used as an indicator of heat stress in animals [50]. In this study, the mineral supplementation and temperature did not significantly affect the MDA and HSP70, while a tendency for an increase in SOD levels was observed in HOM under heat and recovery conditions. This suggests that high concentrations of organic minerals are beneficial in alleviating the side effects of HS.

No significant differences were observed in pH and NH_3_-N according to temperature changes or mineral supply, which explains why the supply of high concentrations of organic minerals did not negatively affect rumen fermentation. HS decreases the proportion of acetate in the rumen, and increases butyrate proportion and propionate concentration [51, 52]. In the present study, non-significant differences in the molar proportions of acetate, propionate, and butyrate were recorded, which might be due to the short-term HS conditions.

In Holstein rumen microbiota under heat stress, the increase in Fibrobacteres and the soluble carbohydrate-digesting bacterium *Treponema* and the decrease in the acetate-producing bacteria Actinobacteria were consistent with previous studies [53, 54]. Additionally, the relative abundance of *Christensenella* increased as the mineral concentration increased under HS conditions. According to Hu et al. [55] and Correia Sales et al. [56], *Christensenella* mainly affects rumen energy metabolism, and becomes relatively abundant when high-energy feed is consumed. This suggests that the addition of organic minerals may alter the composition of rumen bacterial communities. Although the overall rumen microbiome was not influenced by mineral supplementation, a significant alteration was observed between the HS and recovery periods, which is consistent with earlier studies [57].

## Conclusion

A high concentration of organic mineral supplementation during HS reduced the cortisol concentration and increased the total antioxidant status in Holstein bull calves without altering the overall rumen microbiota. Overall, these results suggest that the side effects of heat stress can be alleviated by adding HOM.

## Data Availability

The datasets analyzed in this study are available from the corresponding author upon request.

## Abbreviations

A:P: Acetate:propionate
ADF: Acid detergent fiber
ADG: Average daily gain
AST: Aspartate aminotransferase
ASV: Amplicon sequence variants
BUN: Blood urea nitrogen
Ca: Calcium
CHOL: Cholesterol
Con: Control
DB: Data base
DM: Dry matter
DMI: Dry matter intake
DNA: Deoxyribonucleic acid
ELISA: Enzyme-linked immunosorbent assay
FBW: Final body weight
Fe: Iron
FE: Feed efficiency
gDNA: Genomic deoxyribonucleic acid
Glu: Glucose
GPX: Glutathione peroxidase
HOM: High-concentration organic minerals
HPLC: High-performance liquid chromatography
HS: Heat stress
HSP: Heat shock protein
IBW: Initial body weight
ICP-MS: Inductively coupled plasma mass spectrometry
IM: Inorganic minerals
MDA: Malondialdehyde
Mg: Magnesium
Mn: Manganese
NDF: Neutral detergent fiber
NH_3_-N: Ammonia nitrogen
OM: Organic minerals
OSI: Oxidative stress index
P: Phosphorus
PCoA: Principal coordinates analysis
rH: Relative humidity
ROS: Reactive oxygen species
rRNA: Ribosomal ribonucleic acid
RT: Rectal temperature
Se: Selenium
SOD: Superoxide dismutase
TAS: Total antioxidant status
TBIL: Total bilirubin
THI: Temperature humidity index
TOS: Total oxidant status
TP: Total protein
UV: Ultraviolet rays
VFA: Volatile fatty acid
Zn: Zinc
